# ^99m^Tc-Labeled HYNIC-DAPI Causes Plasmid DNA Damage with High Efficiency

**DOI:** 10.1371/journal.pone.0104653

**Published:** 2014-08-06

**Authors:** Joerg Kotzerke, Robert Punzet, Roswitha Runge, Sandra Ferl, Liane Oehme, Gerd Wunderlich, Robert Freudenberg

**Affiliations:** University Hospital/Faculty of Medicine Carl Gustav Carus, Technische Universität Dresden, Department of Nuclear Medicine, Dresden, Germany; RIKEN Advanced Science Institute, Japan

## Abstract

^99m^Tc is the standard radionuclide used for nuclear medicine imaging. In addition to gamma irradiation, ^99m^Tc emits low-energy Auger and conversion electrons that deposit their energy within nanometers of the decay site. To study the potential for DNA damage, direct DNA binding is required. Plasmid DNA enables the investigation of the unprotected interactions between molecules and DNA that result in single-strand breaks (SSBs) or double-strand breaks (DSBs); the resulting DNA fragments can be separated by gel electrophoresis and quantified by fluorescent staining. This study aimed to compare the plasmid DNA damage potential of a ^99m^Tc-labeled HYNIC-DAPI compound with that of ^99m^Tc pertechnetate (^99m^TcO_4_^−^). pUC19 plasmid DNA was irradiated for 2 or 24 hours. Direct and radical-induced DNA damage were evaluated in the presence or absence of the radical scavenger DMSO. For both compounds, an increase in applied activity enhanced plasmid DNA damage, which was evidenced by an increase in the open circular and linear DNA fractions and a reduction in the supercoiled DNA fraction. The number of SSBs elicited by ^99m^Tc-HYNIC-DAPI (1.03) was twice that caused by ^99m^TcO_4_^−^ (0.51), and the number of DSBs increased fivefold in the ^99m^Tc-HYNIC-DAPI-treated sample compared with the ^99m^TcO_4_^−^ treated sample (0.02 to 0.10). In the presence of DMSO, the numbers of SSBs and DSBs decreased to 0.03 and 0.00, respectively, in the ^99m^TcO_4_^–^ treated samples, whereas the numbers of SSBs and DSBs were slightly reduced to 0.95 and 0.06, respectively, in the ^99m^Tc-HYNIC-DAPI-treated samples. These results indicated that ^99m^Tc-HYNIC-DAPI induced SSBs and DSBs via a direct interaction of the ^99m^Tc-labeled compound with DNA. In contrast to these results, ^99m^TcO_4_^−^ induced SSBs via radical formation, and DSBs were formed by two nearby SSBs. The biological effectiveness of ^99m^Tc-HYNIC-DAPI increased by approximately 4-fold in terms of inducing SSBs and by approximately 10-fold in terms of inducing DSBs.

## Introduction

The study of cellular DNA damage by various chemical and physical events is hampered by the existence of effective repair systems. Moreover, cellular and nuclear membranes are barriers that are difficult to overcome. Plasmid DNA is a model that enables the investigation of unprotected interactions between molecules and DNA that result in single-strand breaks (SSBs) or double-strand breaks (DSB); the resulting fragments can be separated by gel electrophoresis and quantified by fluorescent staining. Auger electron-emitting radiotracers can be implanted directly into DNA via the incorporation of radiolabeled thymidine analogues or by indirect DNA labeling with radioactive dyes to study the influence of geometry [1], [2] or to characterize different isotopes of the same element [3].

Most data have been obtained using ^125^I-labeled radiotracers. Recently, ^99m^Tc has become an interesting candidate for imaging and radiotherapy. Haefliger et al. demonstrated the induction of DSBs by Auger electrons from ^99m^Tc complexes with DNA-binding ligands using gel electrophoresis; however, the damage was not quantified under consideration of the absorbed dose [4]. Santos-Cuevas et al. demonstrated by microscopy and with proliferation assays that ^99m^Tc-labeled bombesin effectively trafficked to the nucleus in prostate and breast cancer cells [5]. Schipper et al. reported that ^99m^Tc and ^131^I accumulated in NIS-positive neuroendo­crine tumor cells in vitro and in a preclinical model, suggesting the potential for both imaging and therapy [6]. Kriehuber et al. investigated cell survival, the induction of apoptosis and micronucleus formation in SCL-II cells after exposure to the Auger electron emitter ^99m^Tc [7], but cellular uptake was not sufficient to ascertain the radiobiological effectiveness. Kotzerke et al. compared the uptake of ^99m^Tc versus ^211^At in NIS-positive rat thyroid cells [8], Wendisch et al. investigated the clonogenic survival after intracellular uptake of ^99m^Tc vs. ^131^I [9], whereas Freudenberg et al. compared ^99m^Tc to ^188^Re. [10]. Because nuclear uptake was negligible, DNA damage by low-energy Auger electrons could not be detected. Wunderlich et al. increased the intracellular accumulation of ^99m^Tc by preincubation with Sn-complexes [11]. Recently, the importance of low-energy Auger and conversion electrons was reported by Cambien et al., who investigated ^99m^TcO_4_^−^ mediated thyroid stunning in vivo [12].

One possibility for transporting radioisotopes into the nucleus is to use radiolabeled fluorescent dyes [13], [14]. DAPI (4′,6-diamidino-2-phenylindole) is a DNA-specific fluorescent probe that interacts with DNA by inserting into the minor grooves of AT-rich DNA sequences [15]. In our study, DAPI was labeled with ^99m^Tc via a HYNIC (6-hydrazinonicotinic) linker. We characterized the damage to plasmid DNA caused by the Auger electron emitter ^99m^Tc using quantitative gel electrophoresis in the presence or absence of the radical scavenger DMSO and compared the radiobiological effectiveness with that of unbound ^99m^Tc-pertechnetate.

## Methods

### Preparation of ^99m^Tc-HYNIC-DAPI

**Figure 1:**
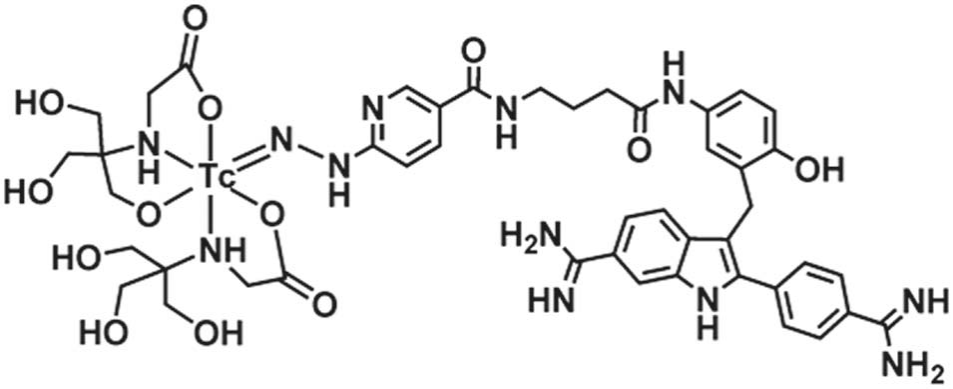
Supposed structure of [^99m^Tc]Tc(Tricin)_n_HYNIC-DAPI (^99m^Tc-HYNIC-DAPI) with HYNIC as monodentate ligand.

Chemicals were purchased from Sigma-Aldrich Chemical Company (Taufkirchen, Germany) unless otherwise stated. The following procedure was used to radiolabel 6-hydrazinonicotinamide (HYNIC)-conjugated DAPI (N-(4-(3-((6-carbamimidoyl-2-(4-carbamimidoylphenyl)-1H-indol-3-yl)methyl)-4-hydroxyphenylamino)-4-oxobutyl)-6-(2-(2,2,2-trifluoracetoxy)hydrazinyl)nicotinamide) (HYNIC-DAPI; ABX, Radeberg, Germany) with ^99m^Tc (Figure 1). Briefly, 0.2 mL of an argon-purged N-(2-hydroxy-1,1-bis(hydroxymethyl)ethyl)glycine (tricine) coligand solution (200 mg tricine (0.11 mmol)/10 mL deionized water plus 1.5 mg tin(II)chloride dihydrate (0.53 mmol)) was added to 5 µg (7.00*10^−9^ mol) of HYNIC-DAPI. After 5 min, 1 mL of ^99m^Tc generator eluate (1.5–2 GBq, Mallinckrodt Pharmaceuticals, Neustadt, Germany) was added, and the mixture was lightly shaken for 30–60 min at room temperature. The radiochemical purity of [^99m^Tc]Tc(Tricin)_n_HYNIC-DAPI (^99m^Tc-HYNIC-DAPI) was determined by analytical HPLC (Chromolith Performance RP-18e 100-4.6 mm, Merck, Darmstadt, Germany) using a linear solvent gradient of 4–15% B over 20 min (A = H_2_O with 0.05% TFA; B = acetonitrile with 0.05% TFA) and a flow rate of 1.4 mL/min. The same HPLC conditions were used to determine the stability of the radioligand solution after storing it at room temperature.

### Plasmid DNA

The pUC19 plasmid (2686 base pairs; MW 1.75*10^6^ Daltons) originating from E. coli ER2272 was purchased from New England Biolabs, Ipswich, UK.

DNA stock solutions were diluted in TE buffer (10 mM Tris-HCl and 1 mM EDTA, pH 7.5) to a final concentration of 0.1 µg/µL and were stored at −20°C. Only samples containing > 95% closed circular DNA were used. pUC19 was digested with BamHI (Invitrogen, Karlsruhe, Germany) to obtain linear DNA.

### Irradiation Procedure

The necessary samples, each containing 200 ng plasmid DNA at 0.1 µg/µL and various amounts of the radioactive solutions, were placed in microtubes (Eppendorf, Hamburg, Germany). One radiotracer-free microtube served as the control.

^99m^Tc-HYNIC-DAPI was added to the pUC19 plasmid solutions (0.1–25 MBq/20 µL = 5.0–1250 MBq/mL) in a total sample volume of 20 µL.

^99m^Tc-pertechnetate solutions were adjusted to the same radioactivity as the ^99m^Tc-HYNIC-DAPI solutions and were also added to the plasmid samples. In an additional test series, 0.2 M DMSO (final concentration) was added to the radiotracer solutions. Each sample was separated into two 10 µL aliquots to enable the accumulation of radiation dose for 2 h at 4°C or for 24 h at −20°C.

After irradiation, 10 µL of each irradiated DNA solution was mixed with 1.25 µL of loading buffer. The mixture was pipetted into the wells of a 1.4% agarose gel in TAE buffer. The samples were run at 4 V/cm for 120 min at 6°C. After electrophoresis, the closed circular (supercoiled), open circular and linear plasmid DNA fractions were identified based on mobility differences in the gel. The gel was stained with ethidium bromide (0.5 µg/mL) for 30 min, and then the different forms of DNA were visualized using an UV transilluminator (Diana III Digital Imaging System, Raytest, Straubenhardt, Germany). The gel was imaged using a charge-coupled device (CCD) camera. The relative amount of DNA in each conformation was quantified by integrating the corresponding intensity using the open-source platform software Fiji [16]. To quantitate the DNA-bound ^99m^Tc-HYNIC-DAPI activity, the DNA bands corresponding to the supercoiled, linear and open circular forms were excised, and the gel pieces were analyzed using a gamma counter (Cobra II Auto-Gamma, Perkin Elmer life sciences).

### Dosimetry

The dose calculations were performed using the Geant4 Monte Carlo toolkit. Although the real shape of the liquid volume in the microtube is similar to an inverse cone with a spherical apex, the target volume was considered to be a sphere with a 10-µL volume composed of water. The decay sites were chosen to be randomly distributed inside the sphere, corresponding to a homogeneous activity distribution. The emission spectrum of ^99m^Tc was taken from Howell [17] (Table 1). An S value for self-irradiation was obtained by simulating 1 million trajectories and scoring the energy deposition inside the target volume. The total dose was calculated as the product of S and the time-integrated activity. It must be noted that no plasmid binding of radioactivity was considered, because that would require nanodosimetric calculations for the plasmid DNA.

**Table 1:** Emission Spectrum of ^99m^Tc (Data from Howell [17]).

Nomenclature	Average energy (keV)	Yield per decay	Range (µm)
gamma2	141	88.9%	
IC 1 M,N	1.82	99.1%	0.165
IC 2 K	119	8.43%	193
IC 2 L	137	1.36%	244
IC 2 M,N	140	0.37%	251
IC 3 K	122	0.59%	199
IC 3 L	140	0.25%	250
Auger KLL	15.3	1.26%	5.57
Auger KLX	17.8	0.47%	7.25
CK LLX	0.043	1.93%	0.003
Auger LMM	2.05	8.68%	0.199
Auger LMX	2.32	1.37%	0.241
Auger LXY	2.66	0.12%	0.3
CK MMX	0.116	74.7%	0.006
Auger MXY	0.226	110%	0.011
CK NNX	0.033	198%	0.002
X Kalpha1	18.4	3.89%	
X Kalpha2	18.3	2.17%	
X Kbeta1	20.6	0.76%	
X Kbeta2	21.0	0.15%	
X Kbeta3	20.6	0.27%	
X L	2.45	0.49%	
X M	0.236	0.12%	

### Calculation of SSBs and DSBs

The mean number of single-strand breaks (*N_SSB_*) and double-strand breaks (*N_DSB_*) per plasmid were calculated based on the theoretical considerations published by Cowan et al. [18]. *N_DSB_* was estimated based on the relative proportion *L* of linear plasmids:
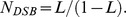



*N_SSB_* was calculated using the fraction *SC* of supercoiled plasmids:
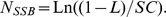



## Results

### Preparation of ^99m^Tc-HYNIC-DAPI

**Figure 2:**
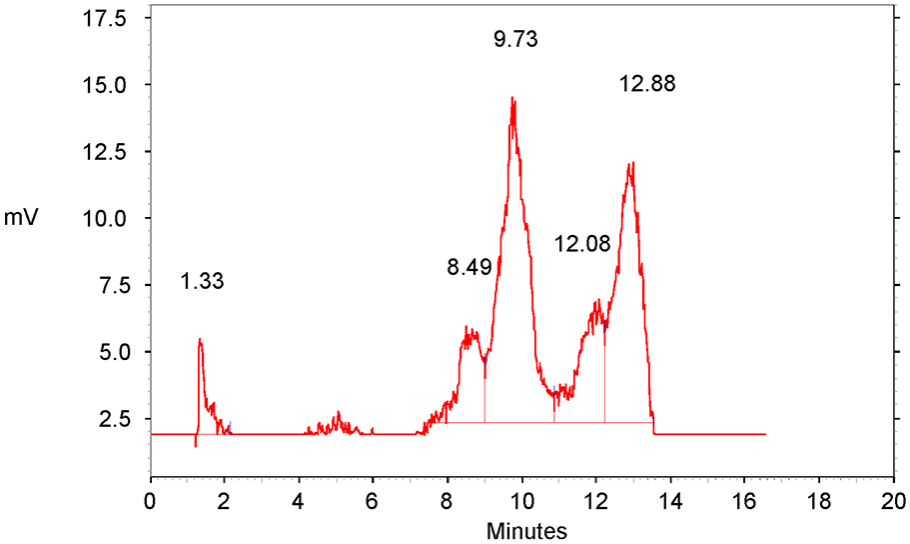
RP-HPLC Chromatogram of ^99m^Tc-HYNIC-DAPI. HPLC trace of the radioactivity 60(3.6%). The signals from 8.49 to 12.88 min represent different ^99m^Tc-HYNIC-DAPI derivatives (96.4%). After 12 h, the peak at 1.33 min increased to 100%.

The yield and stability of ^99m^Tc-HYNIC-DAPI in the reaction solution (saline) were determined using reverse-phase HPLC (Figure 2). The reaction yield of ^99m^Tc-HYNIC-DAPI increased to >95% in one hour. ^99m^Tc-HYNIC-DAPI exhibited low stability in the kit solution (50% at 6 h, <1% after 12 h) at room temperature. Therefore, the peak at a shorter retention time (1.33 min) in the HPLC trace, representative of molecules such as pertechnetate, increased from <5% at 1 h to approximately 100% at 12 h.

### Dose calculations

The S value for the dose calculations was 2.53*10^−10^ Gy/(Bq s) inside a sphere with a 10-µL volume and a homogeneous activity distribution. Therefore, incubating plasmids with 1000 MBq/mL of ^99m^Tc in 10 µL for 2 h yields a dose of 16.3 Gy. Irradiation for 24 h produces a total dose of 74 Gy.

### Strand breaks in pUC19 plasmid DNA

**Figure 3:**
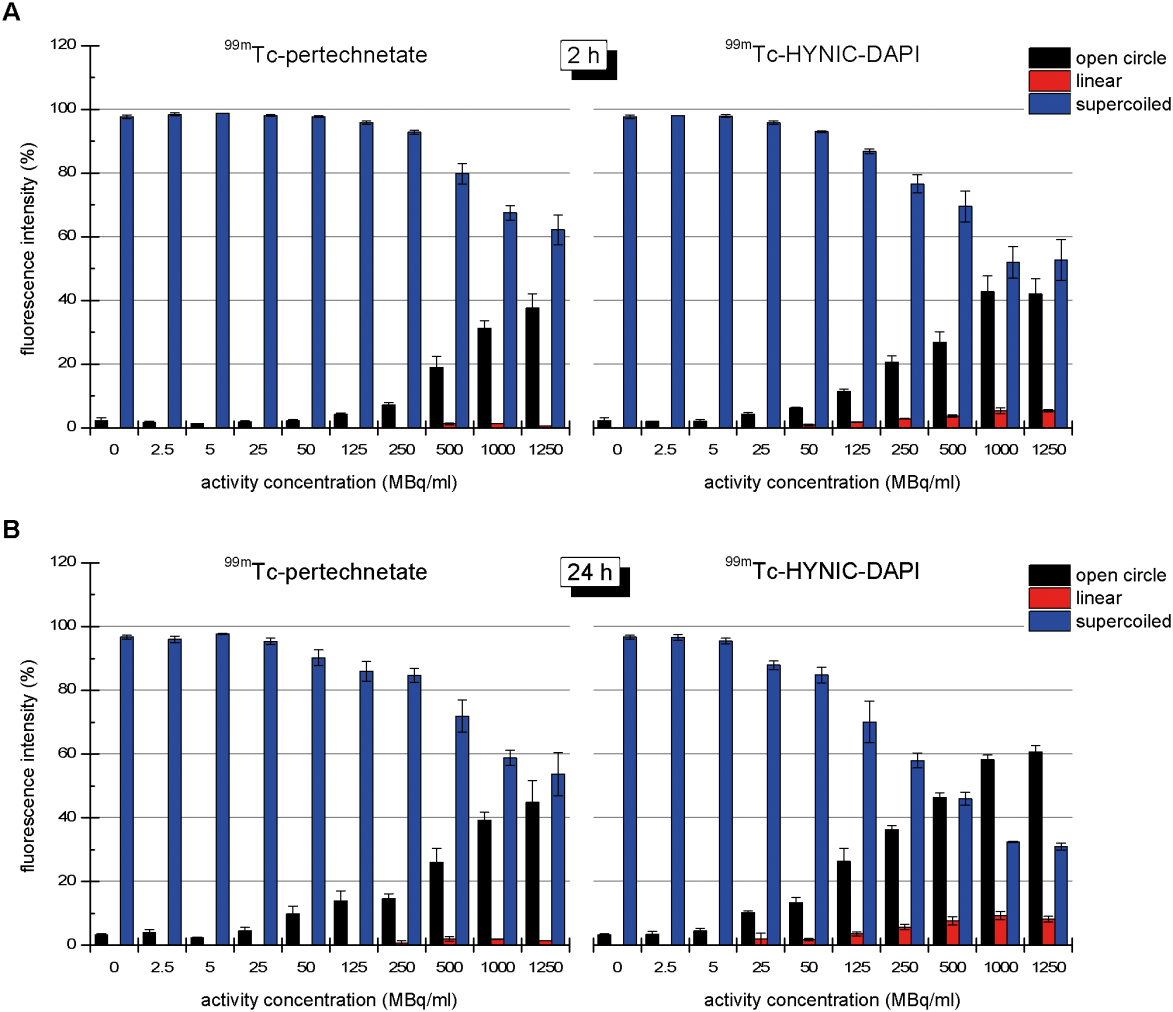
Dose-Response of ^99m^Tc-pertechnetate and ^99m^Tc-HYNIC-DAPI. Dose-dependent decrease in supercoiled DNA exposed to ^99m^Tc-pertechnetate or ^99m^Tc-HYNIC-DAPI for 2 (A) or 24 hours (B). The dose-dependent increase in open circular DNA was similar for both radiotracers, but the fraction of linear DNA was greater in the presence of ^99m^Tc-HYNIC-DAPI compared with ^99m^Tc-pertechnetate.

The fractions of the different conformations of plasmid DNA (open circular, linear and supercoiled) that were observed after 2 or 24 h of irradiation with ^99m^Tc-pertechnetate or ^99m^Tc-HYNIC-DAPI are presented in Figure 3. For both compounds, an increase in applied activity enhanced the plasmid damage, resulting in an increase in the open circular and linear fractions and a decrease in the supercoiled fraction. The DNA damage was more extensive after 24 h of irradiation than after 2 h of irradiation. Compared with ^99m^Tc-pertechnetate, the radiotoxicity of ^99m^Tc-HYNIC-DAPI was obviously higher. For example, after a 24 h irradiation with 1000 MBq/mL of ^99m^Tc-pertechnetate or ^99m^Tc-HYNIC-DAPI, the open circular DNA fractions were 39.3% and 58.2%, respectively, and the linear DNA fractions were 1.9% and 9.3%, respectively.

Based on the measured fluorescence intensities, the numbers of SSBs and DSBs were calculated (Table 2). In the example above, the number of SSBs created by ^99m^Tc-HYNIC-DAPI (1.03) was twice that elicited by ^99m^Tc-pertechnetate (0.51). The number of DSBs increased fivefold (from 0.02 to 0.10) in the ^99m^Tc-HYNIC-DAPI-treated samples compared with the ^99m^Tc-pertechnetate-treated samples. The ratio of the number of SSBs to the number of DSBs decreased from 25 for ^99m^Tc-pertechnetate to 10 for ^99m^Tc-HYNIC-DAPI.

**Table 2:** Mean numbers of DSBs and SSBs induced by ^99m^Tc pertechnetate and ^99m^Tc-HYNIC-DAPI in the absence or presence of DMSO.

	Activity(MBq/mL)	− DMSO				+ DMSO			
		^99m^Tc-pertechnetate		^99m^Tc-HYNIC-DAPI		^99m^Tc-pertechnetate		^99m^Tc-HYNIC-DAPI	
		SSBs	DSBs	SSBs	DSBs	SSBs	DSBs	SSBs	DSBs
2 h	0	0.02	0.00	0.02	0.00				
	2.5	0.02	0.00	0.02	0.00				
	5	0.01	0.00	0.02	0.00				
	25	0.02	0.00	0.04	0.00				
	50	0.02	0.00	0.06	0.01			0.05	0.00
	125	0.04	0.00	0.12	0.02			0.07	0.01
	250	0.07	0.00	0.24	0.03			0.16	0.02
	500	0.21	0.01	0.33	0.04			0.24	0.02
	1000	0.38	0.01	0.60	0.06	0.02	0.00	0.46	0.02
	1250	0.47	0.01	0.59	0.06	0.02	0.00	0.55	0.03
24 h	0	0.03	0.00	0.03	0.00				
	2.5	0.04	0.00	0.03	0.00				
	5	0.02	0.00	0.05	0.00				
	25	0.05	0.00	0.11	0.02				
	50	0.10	0.00	0.15	0.02			0.11	0.01
	125	0.15	0.00	0.32	0.04			0.18	0.02
	250	0.16	0.01	0.49	0.06			0.47	0.04
	500	0.31	0.02	0.70	0.08			0.52	0.04
	1000	0.51	0.02	1.03	0.10	0.03	0.00	0.95	0.06
	1250	0.61	0.02	1.09	0.09	0.03	0.00	1.37	0.08

To assess the chemotoxicity of unlabeled HYNIC-DAPI, we performed experiments with an identical setup using the same ligand concentrations as those used for ^99m^Tc-HYNIC-DAPI. The results revealed that supercoiled plasmid DNA remained completely intact throughout all the incubations in the presence or absence of DMSO. Thus, no chemotoxic effects were observed (data not shown).

**Figure 4:**
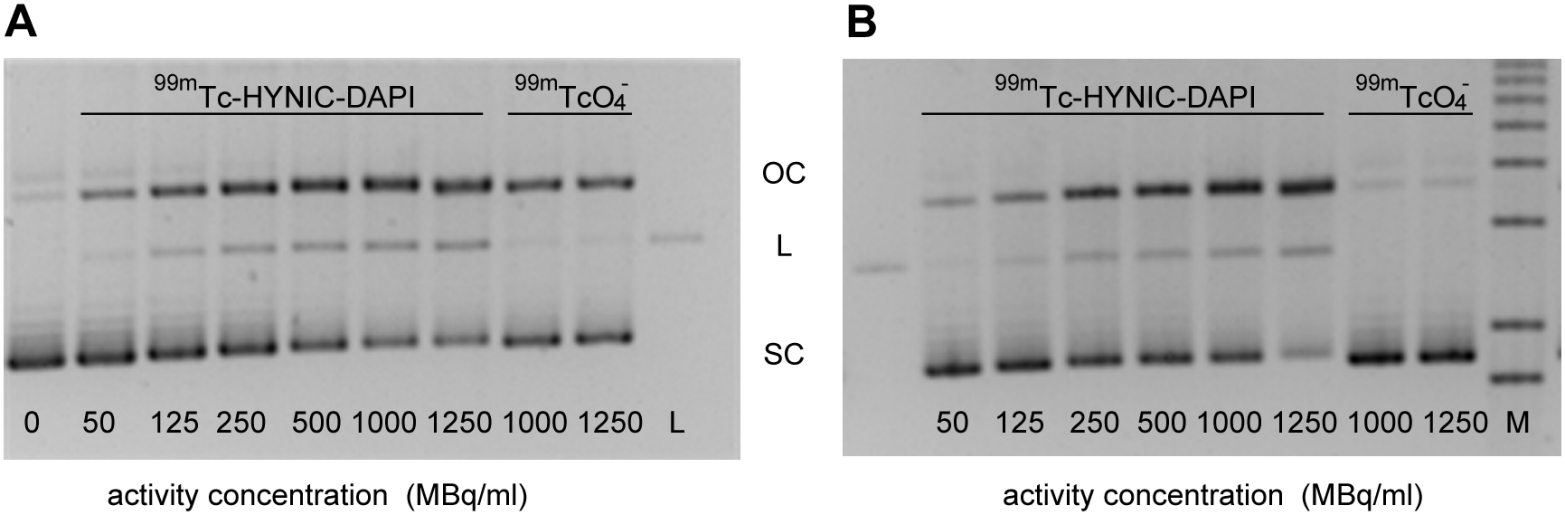
Example images of agarose gel indicating the effect of DMSO. Representative images of agarose gels in the absence (A) or presence (B) of DMSO. The plasmid DNA samples were irradiated for 24 h with 50–1250 MBq/mL ^99m^Tc-HYNIC-DAPI. The influence of DMSO on DNA damage (OC: open circular, L: linear, SC: super coiled plasmid DNA fraction) caused by ^99m^Tc-HYNIC-DAPI was compared with that elicited by ^99m^Tc-pertechnetate (^99m^TcO_4_^−^). Notably, the formation of open circular and linear DNA in response to ^99m^Tc-pertechnetate could be prevented by DMSO. The first lane is a non-irradiated plasmid sample, L is a non-irradiated linear plasmid, and M is the marker. The DNA bands at the bottom represent supercoiled DNA.

**Figure 5:**
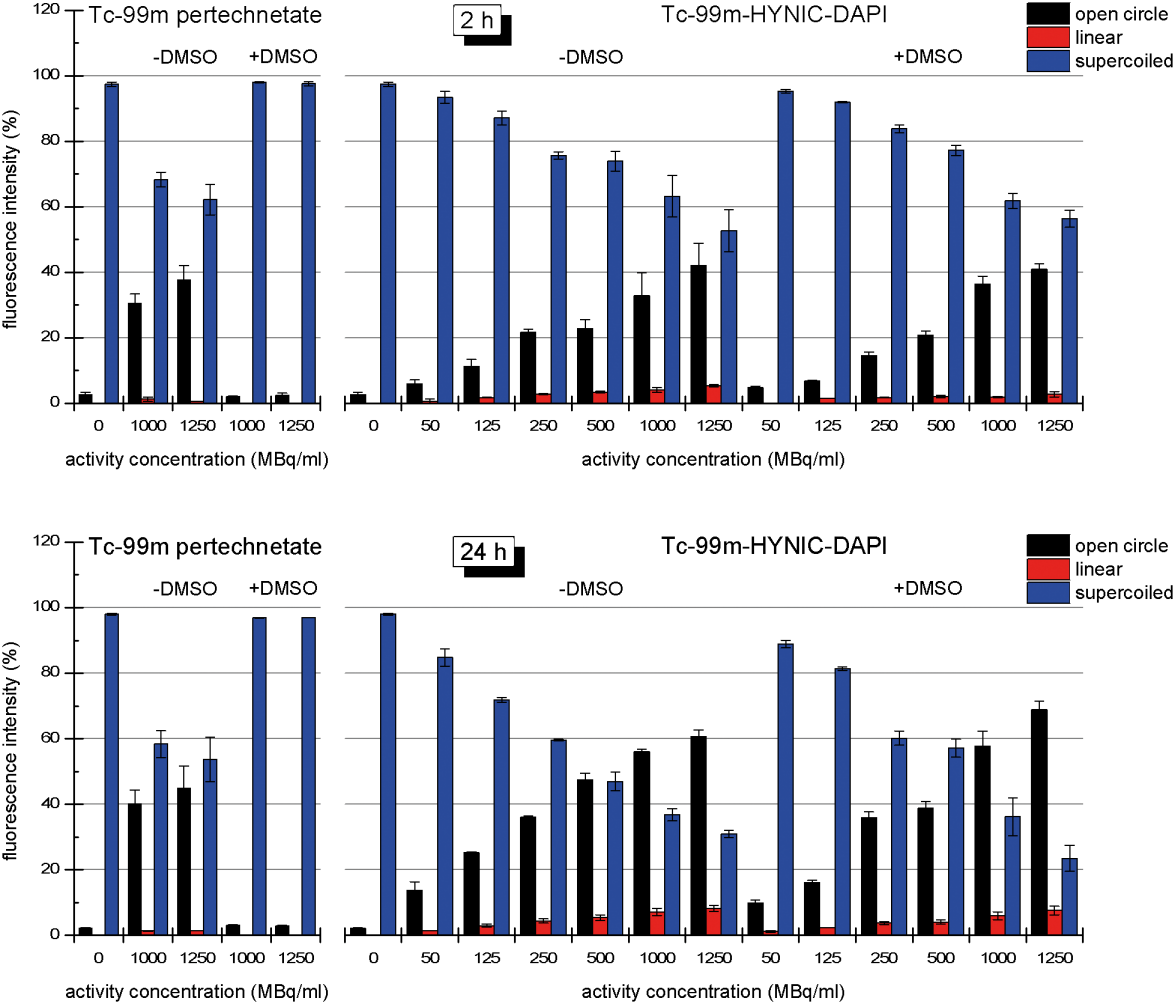
Effect of DMSO on plasmid damage. The influence of the radical scavenger DMSO on the DNA conformational changes caused by ^99m^Tc-pertechnetate and ^99m^Tc-HYNIC-DAPI after 2 (A) or 24 h (B) of irradiation. The formation of open circle and linear DNA in response to ^99m^Tc-pertechnetate was nearly completely suppressed in the presence of DMSO. These data indicated that the plasmid damage by ^99m^Tc-pertechnetate was primarily caused by radicals. There was no noticeable effect of DMSO in the ^99m^Tc-HYNIC-DAPI-treated samples, indicating that the plasmid damage was caused by direct interactions between the radioactive compound and the DNA.

**Figure 6:**
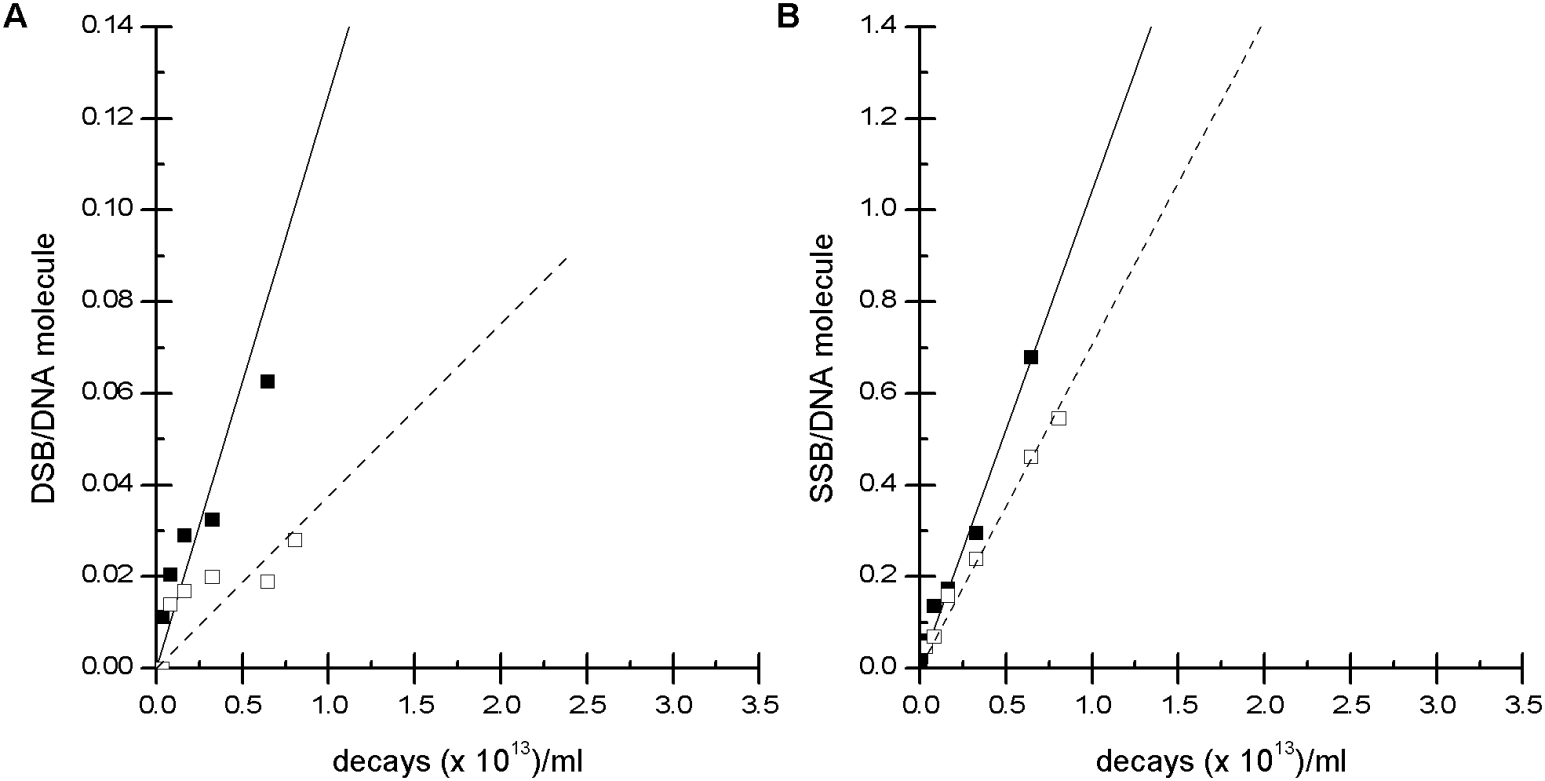
Number of DSBs and SSBs per DNA molecule. Analysis of the mean number of DSBs (A) and SSBs (B) per plasmid molecule as a function of ^99m^Tc-HYNIC-DAPI decays during a 2 h irradiation. The numbers of DSBs and SSBs were calculated based on the fluorescence intensity of the linear and supercoiled DNA fractions in the presence (□) or absence (▪) of DMSO.

To verify the contribution of reactive oxygen species to the formation of SSBs and DSBs, additional experiments were performed in the absence or presence of 0.2 M DMSO. Irradiation of plasmid DNA with ^99m^Tc-HYNIC-DAPI produced similar effects in the absence or presence of DMSO (Figures 4 and 5). For example, the open circular and linear fractions did not noticeably change. After 24 h, 1000 MBq/mL of ^99m^Tc-HYNIC-DAPI resulted in (56.0 ±1.1)% open circular DNA in the absence of DMSO and (57.8±6.4)% open circular DNA in the presence of DMSO. The corresponding linear fractions were (7.2±1.6)% and (6.0±1.7)%, respectively. These results demonstrated that SSBs and DSBs were not markedly reduced by DMSO, suggesting that direct interactions between the ^99m^Tc-labeled compound and DNA predominated. In contrast to these results, the number of SSBs induced by 1000 MBq/mL ^99m^Tc-pertechnetate in the absence of DMSO was significantly reduced from 40% open circular DNA to only 3.0%. Furthermore, the formation of DSBs was completely prevented when the ^99m^Tc-pertechnetate irradiation occurred in the presence of DMSO, as indicated by the absence of a linear DNA fraction (Figure 5). The dependence of SSBs and DSBs on the total number of disintegrations in the presence or absence of DMSO is illustrated in Figure 6.

### Estimation of plasmid-bound activity

**Figure 7:**
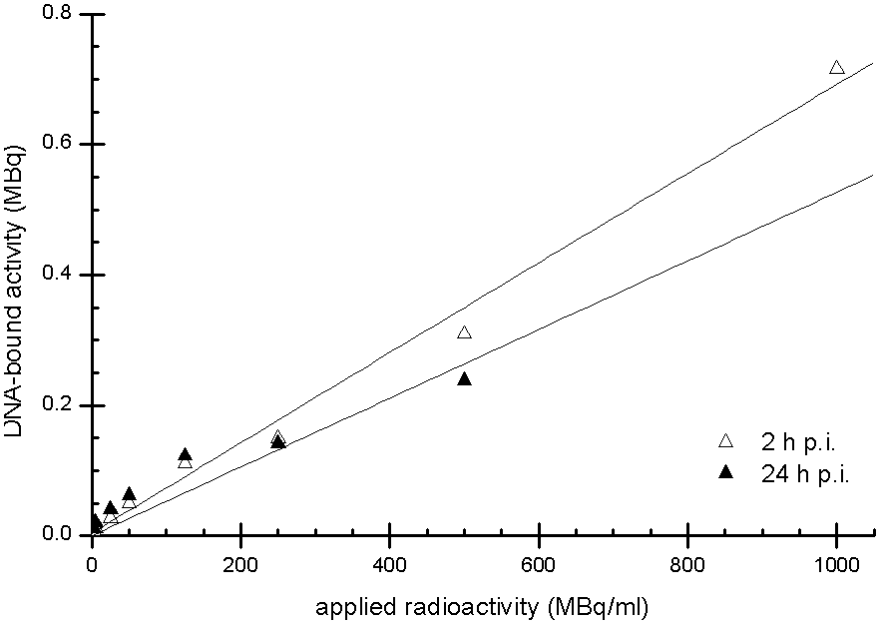
Measurement of the Plasmid DNA-bound radioactivity. The measurements were performed using a gamma counter, and the activity was decay-corrected based on the application time. The data were obtained from a single experiment. A good linear correlation was observed between the applied activity and the bound activity.

**Figure 8:**
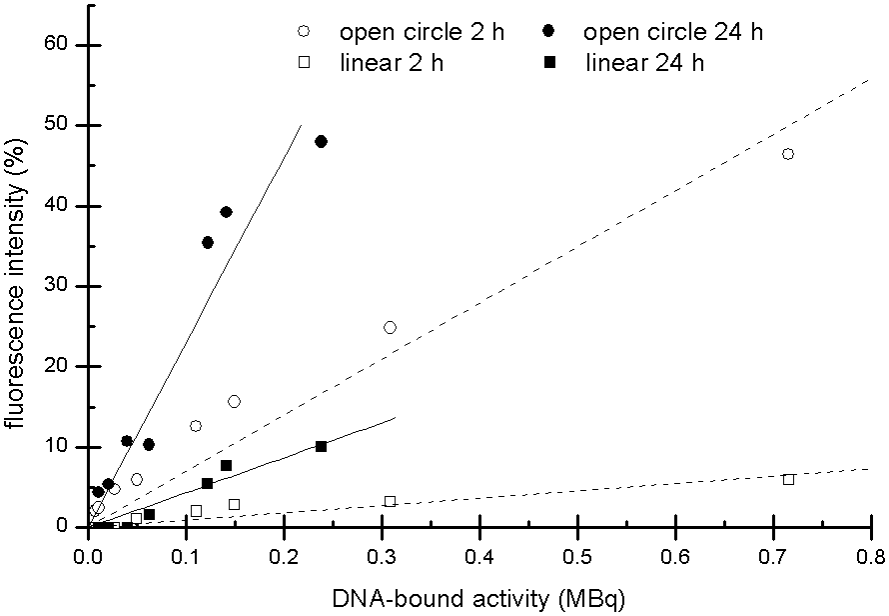
Fluorescence intensity with respect to DNA-bound activity. Dependence of the relative fluorescent intensity of open circle and linear plasmid DNA on the DNA-bound activity. A good correlation was observed between the activity and the radiation damage. The higher fluorescent intensity after the 24 h incubation time was due to the increased number of radioactive decays.

After quantifying the DNA damage by ethidium bromide staining and image processing, the DNA bands representing each plasmid conformation (supercoiled, open circular and linear) were excised, and the DNA-bound activity in the ^99m^Tc-pertechnetate and ^99m^Tc-HYNIC-DAPI samples was measured using a gamma counter. The bound activity was quantitated using decay-corrected values. ^99m^Tc-pertechnetate-irradiated plasmid DNA exhibited a low amount of bound activity (<0.005 MBq) that was independent of the administered activity. In contrast, ^99m^Tc-HYNIC-DAPI demonstrated a correlation between applied activity and DNA-bound activity, reaching a maximum value of 0.72 MBq in the sample dosed with 1000 MBq/mL (Figure 7). When plotting the bound activity against the fractions of open circular DNA or linear DNA (Figure 8), linear correlations were apparent. These correlations indicated that increased plasmid damage resulted from increased ^99m^Tc-HYNIC-DAPI accumulation.

## Discussion

DAPI is a DNA-specific probe that forms a fluorescent complex by inserting into the minor groove of AT-rich DNA sequences [15]. The HYNIC linker is widely used for labeling bioactive substances with ^99m^Tc without a loss of avidity [19]. We found that unlabeled HYNIC-DAPI was not chemotoxic at the tested low concentrations (data not shown).

The plasmids were incubated with ^99m^Tc-HYNIC-DAPI in excess of unlabeled DAPI, which can be explained by stoichiometric calculations. Radiochemistry labeling was performed using 1500 MBq ^99m^Tc and 5 µg HYNIC-DAPI. HYNIC-DAPI has a molar mass of 943 g/mol, which corresponds to a ratio of 4.7*10^13^ atoms of ^99m^Tc to 3.2*10^15^ molecules of HYNIC-DAPI. Assuming 100% turnover, the reaction would yield 1 labeled ^99m^Tc-HYNIC-DAPI molecule per 67 non-radioactive HYNIC-DAPI molecules. Here, the term non-radioactive refers to HYNIC-DAPI that was unlabeled or that was labeled with ^99^Tc, which is considered non-radioactive because of its long half-life (2.1*10^5^ years). Two hundred nanograms of pUC19 plasmid DNA contain 6.9*10^10^ plasmid molecules. When incubating 1 MBq ^99m^Tc-HYNIC-DAPI with 200 ng of pUC19 plasmid DNA, 0.45 ^99m^Tc-HYNIC-DAPI molecules would bind to each plasmid molecule on average.

^99m^Tc-pertechnetate bound to plasmid DNA to a much lesser extent (by approximately 100-fold less, data not shown), suggesting that the binding of ^99m^Tc-HYNIC-DAPI to DNA was a specific event. DAPI molecules bind to double-stranded DNA in the minor groove. The distance between two minor grooves is 10 base pairs; therefore, we assumed that there were 270 binding sites per plasmid for ^99m^Tc-HYNIC-DAPI. Considering the competition by non-radioactive HYNIC-DAPI, a maximum of 4 ^99m^Tc-HYNIC-DAPI molecules could bind to each pUC19 plasmid DNA on average. For our labeling procedure, 4 ^99m^Tc-HYNIC-DAPI molecules per plasmid corresponded to the application of 10 MBq (500 MBq/mL) of ^99m^Tc-HYNIC-DAPI. However, our data did not demonstrate the saturation of plasmid damage at higher activities (Figure 3), indicating that not every available HYNIC-DAPI molecule bound to plasmid DNA.

The DNA damage caused by unbound ^99m^Tc-HYNIC-DAPI was expected to be comparable to that caused by ^99m^Tc-pertechnetate. In total, 1000 MBq/mL of unbound, but homogeneously distributed, ^99m^Tc in a volume of 10 µL leads to an absorbed dose of 74 Gy within 24 h. According to Table 2, this dose produced 0.5 SSBs and 0.02 DSBs on average in the absence of DMSO (24 h, 1000 MBq/mL, ^99m^Tc-pertechnetate). In the presence of DMSO, the formation of SSBs and DSBs was completely prevented (0.03 SSBs, no DSBs). These data suggested that the DNA damage was radical mediated and an indirect effect of unbound ^99m^Tc.

After 2 h of irradiation, 1000 MBq/mL ^99m^Tc-HYNIC-DAPI produced 60% more (from 0.38 to 0.60) SSBs and 300% more (from 0.013 to 0.056) DSBs compared with unbound ^99m^Tc-pertechnetate. These increases resulted from approximately 0.7 MBq DNA-bound radioactivity (Figure 7).

Within 24 h, 500 MBq/mL of unbound ^99m^Tc (Figure 3) caused 26% of the DNA to adopt an open circular configuration. The same percentage was induced by 125 MBq/mL of ^99m^Tc-HYNIC-DAPI, which corresponds to a 4-fold enhancement. The potential for plasmid linearization was approximately ten times higher for ^99m^Tc-HYNIC-DAPI because 50 MBq/mL yielded 2% linear plasmid, whereas 500 MBq/ml of ^99m^Tc-pertechnetate was required to elicit the same effect. A higher linear plasmid fraction could not be induced by ^99m^Tc-pertechnetate because of limited specific activity.

We did not perform X-ray irradiation; hence, we could not determine the relative biological effectiveness of ^99m^Tc-HYNIC-DAPI and ^99m^TcO_4_^−^. Nevertheless, we compared the biological effectiveness of each compound, which we defined as the ratio of the activities of ^99m^Tc-HYNIC-DAPI versus ^99m^TcO_4_^−^ that caused the same effect. Based on this definition, the biological effectiveness increased by approximately 4-fold in inducing SSBs and by approximately 10-fold in inducing DSBs, which is in the range reported for Auger electrons emitted in close proximity to DNA [20].

The SSBs created by unbound ^99m^Tc-pertechnetate could be prevented by DMSO. In contrast, DSBs and SSBs caused by ^99m^Tc-HYNIC-DAPI could not be prevented by DMSO. This indicated that the impact of the Auger events was direct or indirect with an extremely high local energy transfer that could not be antagonized by DMSO. SSBs are usually assumed to be radical-mediated; however, we demonstrated that the induction of SSBs by DNA-bound ^99m^Tc-HYNIC-DAPI might occur via another mechanism. It is possible that SSBs were also caused by direct interactions between emitted low-energy electrons and plasmid-bound ^99m^Tc-HYNIC-DAPI that merely damaged a single strand. On average, 4.9 electrons are emitted by the decay of ^99m^Tc via internal conversion and gamma emission. This event creates a dramatic charge transfer in the electron shell, which can cause a Coulomb explosion of the molecule, as discussed by Pomplun and Sutmann [21].

The absorbed dose was calculated based on the absorbed energy inside an incubation volume of 10 µL. Thus, no regional heterogeneities, such as DNA-bound activity, were considered. Therefore, the same amount of ^99m^Tc yielded the same absorbed dose regardless of whether ^99m^Tc-pertechnetate or ^99m^Tc-HYNIC-DAPI was used. We expected that the dose to the plasmids was higher with ^99m^Tc-HYNIC-DAPI due to the emission of low-energy electrons. ^99m^Tc emits an average of 4.9 electrons with less than 2.7 keV of energy and a maximum range of 300 nm [17]. However, the term ‘absorbed dose’ is a macroscopic quantity that does not account for stochastic effects. Hence, we did not calculate the absorbed dose on plasmid level because ^99m^Tc-HYNIC-DAPI binding is a random process, as are radioactive decay and the direction of radiation emission.

The ratio of SSBs to DSBs induced by irradiation is approximately 40∶1 per gray of gamma radiation [22]; this ratio decreases with increasing dose because SSBs are converted to DSBs. High LET irradiation induces more DSBs because the high density of radicals increases the possibility of DSBs being generated by chance. We calculated the SSB and DSB yields based on the conversion of supercoiled plasmid DNA into linear DNA. The numbers of SSBs and DSBs per plasmid molecule based on the number of decays over 2 h are plotted in Figure 6. As expected, there was a linear correlation between the number of decays and the number of SSBs and DSBs per plasmid molecule. DNA-bound ^99m^Tc-HYNIC-DAPI produced DNA damage with a SSB:DSB ratio of 10∶1 in the absence of DMSO. Furthermore, 0.64*10^13^ decays/mL induced 0.06 DSBs and 0.68 SSBs per molecule, which corresponded with 1 DSB per 10.2*10^13^ decays/mL. Balagurumoorthy et al. reported values between 5.57*10^13^ and 29.6*10^13^ decays/mL for various ^125^I compounds in the absence of DMSO [2]. The yield of DSBs per decay of ^99m^Tc was calculated as the product of the plasmid concentration and the number of DSBs per decay/mL. Although Balagurumoorthy obtained DSB yields ranging from 0.10 to 0.52 per decay of ^125^I, our data demonstrated a DSB yield of 0.03 per decay of ^99m^Tc. Therefore, our observed number of decays/mL corresponded with the data published by Balagurumoorthy, although the DSB yield for ^99m^Tc appeared to be a tenth of that for ^125^I because of the lower plasmid amount. We irradiated 200 ng of pUC19 plasmid DNA in a volume of 20 µL, which corresponds to 3.44*10^12^ plasmid molecules/mL, whereas Balagurumoorthy used 3.06*10^13^ molecules/mL.

A more reliable calculation of the DSB yield per plasmid-bound decay requires knowledge of the amount of plasmid-bound radioactivity. Furthermore, the radiochemical stability of ^99m^Tc-HYNIC-DAPI will introduce variability in the number of decays per plasmid. The plasmid-bound applied activity varied between 2% and 10% in different experiments. Assuming 5% plasmid-bound applied activity, we calculated 0.5 to 1.5 DSBs per plasmid-bound decay. Humm and Charlton calculated the probability of DSB formation due to DNA-bound Auger electron emission for ^125^I, ^123^I and ^99m^Tc to be 1.1, 0.73 and 0.43, respectively [23].

Several studies have characterized the influence of the distance between the decaying isotope and DNA on DSB yield. Balagurumoorthy et al. synthesized a series of ^125^I-labeled Hoechst derivatives with a minor groove-binding motif and an increasing distance between the radioactive molecule and the binding position (ranging from 10.5 Å to 13.9 Å) [2]. The DSB yield decreased with distance from 0.2 DSBs/decay to 0.025 DSBs/decay, demonstrating the crucial influence of relatively small distances. Although DMSO could not prevent the formation of DSBs at the closest distance, it reduced DSBs by 10-fold at greater distances. Our data did not indicate an effect of DMSO on DNA-bound ^99m^Tc-HYNIC-DAPI. Therefore, we assumed that the radioactive molecule was below the critical distance from the DNA helix, even if stereoisomers could exist.

Walicka et al. investigated the effect of different DMSO concentrations (0.26 to 3 M) on the radiotoxicity of the Auger electron emitter ^125^I [24]. The authors concluded, that the most radiotoxic effects from DNA-incorporated ^125^I were due to indirect mechanisms. In contrast to our experiments Walicka et al. performed experiments using V79 cells, whereas we used plasmids as biological model without any repair or radioprotection by other cellular structures. Additionally, the authors irradiated their cells at −135°C what could be cryotoxic to cells. Balagurumoorthy et al. found that in the absence of DMSO, ^125^IEH-induced DSBs in nicked or linear DNA were caused by both direct and indirect mechanisms, whereas in its presence, DSBs occurred predominantly by direct ionization of DNA [25].

## Conclusion

The direct binding of ^99m^Tc-HYNIC-DAPI to plasmid DNA was demonstrated and quantified. Plasmid-bound ^99m^Tc-HYNIC-DAPI induced SSBs and DSBs with high efficiency. DNA damage could not be prevented by the radical scavenger DMSO, suggesting that the damage was a direct effect. This statement is true for both DSBs and SSBs. The biological effectiveness increased by approximately 4-fold for inducing SSBs and approximately 10-fold for inducing DSBs, which was within the range reported for Auger electrons emitted in close proximity to DNA.
